# Optimal Design of Patient-Specific Total Knee Arthroplasty for Improvement in Wear Performance

**DOI:** 10.3390/jcm8112023

**Published:** 2019-11-19

**Authors:** Yong-Gon Koh, Kyung-Hwan Jung, Hyoung-Taek Hong, Kang-Min Kim, Kyoung-Tak Kang

**Affiliations:** 1 Joint Reconstruction Center, Department of Orthopaedic Surgery, Yonsei Sarang Hospital, 10 Hyoryeong-ro, Seocho-gu, Seoul 06698, Korea; 2 Additive Manufacturing Process R&D Group, Korea Institute of Industrial Technology, Gangneung 25440, Korea; 3 Department of Mechanical Engineering, Yonsei University, 50 Yonsei-ro, Seodaemun-gu, Seoul 03722, Korea

**Keywords:** patient-specific total knee arthroplasty, wear performance, gait cycle, optimal design

## Abstract

Life expectancy is on the rise and, concurrently, the demand for total knee arthroplasty (TKA), which lasts a lifetime, is increasing. To meet this demand, improved TKA designs have been introduced. Recent advances in radiography and manufacturing techniques have enabled the production of patient-specific TKA. Nevertheless, concerns regarding the wear performance, which limit the lifespan of TKA, remain to be addressed. This study aims at reducing the wear in patient-specific TKA using design optimization and parametric three-dimensional (3D) finite-element (FE) modelling. The femoral component design was implemented in a patient-specific manner, whereas the tibial insert conformity remained to be determined by design variables. The gait cycle loading condition was applied, and the optimized model was validated by the results obtained from the experimental wear tests. The wear predictions were iterated for five million gait cycles using the computational model with force-controlled input. Similar patterns for internal/external rotation and anterior/posterior translation were observed in both initial and optimal models. The wear rates for initial and optimal models were recorded as 23.2 mm^3^/million cycles and 16.7 mm^3^/million cycles, respectively. Moreover, the experimental wear rate in the optimal design was 17.8 mm^3^/million cycles, which validated our optimization procedure. This study suggests that tibial insert conformity is an important factor in influencing the wear performance of patient-specific TKA, and it is capable of providing improved clinical results through enhanced design selections. This finding can boost the future development of patient-specific TKA, and it can be extended to other joint-replacement designs. However, further research is required to explore the potential clinical benefits of the improved wear performance demonstrated in this study.

## 1. Introduction

Total knee arthroplasty (TKA) is a standard surgical procedure that is aimed at restoring function, relieving pain, and providing overall satisfaction to patients suffering from knee-joint arthritis [1]. The effects of factors such as changing demographics and body mass index on the demand for TKA have recently been reported to be more significant than those on the demand for total hip arthroplasty [2]. With the increase in life expectancy, patients are relying on TKA to last longer, thereby leading to a pressing need for longevity improvement and revision-rate reduction of TKA. The primary TKA is known for its excellent long-term clinical success rates; however, the dissatisfaction rates among patients are relatively high [3,4]. The common causes for dissatisfaction after TKA include the inability to regain pre-operative function, reduced range of motion (ROM), crepitation, residual pain, and wear [5,6,7]. In recent years, several technologies have been developed for improving the functional results of TKA [7]. For example, computer-assisted surgery improves alignment during TKA. However, all computer-navigated and robotic systems require an additional stage of registration. They incur high costs and consume a considerable amount of time; these are the major problems encountered in low-volume hospitals [8,9]. Patient-specific instruments (PSIs) with goals similar to those of computer navigation (limb alignment and absence of morbidity associated with intramedullary instrumentation) were designed to overcome these problems and simplify the procedure [10]. Some recently conducted studies have suggested an anatomical approach using “patient-specific” or “customized” TKA [5]. In addition, this approach is used together with PSIs as bone cutting is performed using the anatomy of the patient for patient-specific TKA [11]. Patient-specific TKA can address the shortcomings of current off-the-shelf TKA and improve the bony coverage on the tibia and femoral sides [12]. Some believe that these benefits can provide improved satisfaction, reduced cost, shorter hospital stays, and reduced aseptic-loosening rates, which is the most common cause of revision [7,13,14]. However, in current patient-specific TKA, the femoral component is designed based on patient anatomy, whereas the tibial insert is constructed using articular geometry derived from the femoral component [15]. Tibial-insert conformity has been among the main focus points of research based on TKA design [16]. Furthermore, a recent study reported that the tibiofemoral articular surface conformity must be considered carefully in customized patient-specific TKA design because the wear and kinematics can be influenced by tibiofemoral conformity in patient-specific TKA [17]. The effects of wear and osteolysis on long-term survival in TKA are less than those in total hip arthroplasty (THA) [18]. Wear is one of the major causes of late revisions in TKA [6]. Dargahi et al. investigated the optimization of the geometry of a tibial insert knee implant in the sagittal plane with the minimum amount of wear [19]. Willing and Kim designed an optimal TKA using a parametric three-dimensional (3D) finite element (FE) model while considering the wear of the tibial inset [20]. However, they studied conventional TKA and predicted the wear reduction solely by conducting a computational simulation that was not validated by experiments.

Therefore, the aim of this study was to reduce wear in patient-specific TKA using design optimization and the parametric 3D FE model. The gait cycle loading condition was applied, and the optimized model was validated by the results obtained from experimental wear tests. We hypothesized that the tibial insert conformity in the optimal design reduces the wear.

## 2. Material and Methods

### 2.1. Design of Patient Specific TKA

**Figure 1:**
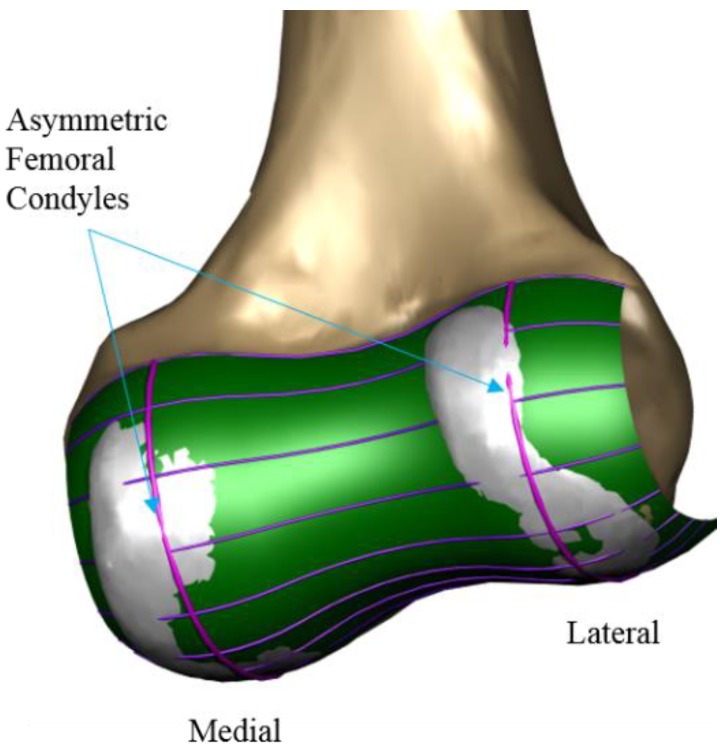
Patient-specific three-dimensional (3D) model design with actual anatomical geometries.

A patient-specific TKA was developed using an existing 3D knee-joint model and the computed tomography (CT) and magnetic resonance imaging (MRI) of a healthy 36-year-old male with a normal knee condition (mechanical femoral tibial angle: varus 1°) [21,22,23,24,25]. The patient-specific TKA model was designed with actual anatomical geometries (Figure 1). CT and MRI were performed using a 64-channel CT scanner (Somatom Sensation 64; Siemens Healthcare; Erlangen, Germany) and a 3T MRI system (Discovery MR750w; GE Healthcare; Chicago, IL, United States), respectively. To develop the 3D model, the 3D reconstruction of the lower extremity was initially performed by segmenting CT and MRI images using Mimics 17.0 (Materialise Ltd., Leuven, Belgium), and combining the positional alignment from each 3D model using Rapidform (3D Systems Korea Inc., Seoul, Korea) [26]. The design procedure of the femoral component was similar to that of our previous patient-specific TKA, and it is briefly described in [17]. The femoral and tibial components were developed using the Unigraphics NX software (Version 7.0; Siemens PLM Software, Torrance, CA, USA). Three patient-specific “J” curves for the trochlear grooves and the medial and lateral condyles from the normal articular anatomy were developed. On the tibial plateau, the outline of the patient’s tibia defines the tibial component geometry. As mentioned previously, the articular geometry of a patient-specific tibial insert design is derived from the femoral component [7,15]. Therefore, the articular conformity of the initial tibial insert was derived from the femoral component.

### 2.2. Development of the Parametric FE Model for Patient-Specific TKA

An existing FE-TKA method was used in this study [17,27]. Patient-specific TKA is composed of three main parts: the femoral component, tibia insert, and tibial component. Rigid body assumptions were applied to both femoral and tibial components. The femoral and tibial components with a high elastic modulus that is approximately 300 times higher than that of the tibial insert were developed as rigid bodies, requiring only surface representation [17,28]. The tibial insert was modeled using tetrahedral elements. The convergence test was performed as described in our previous study [17]. The standard polyethylene (PE) was used for the tibial insert. The standard PE was modeled using isotropic elastic–plastic with a modulus of elasticity of 463 MPa and a Poisson’s ratio of 0.46 as reported by Godest et al. [17]. A friction coefficient of 0.04 was considered between the femoral and tibial components [28]. Overall, in a penalty-based contact model, the contact pressure is computed as a function of the penetration distance of the femoral component into the tibial insert. The development of a parametric FE model is challenging because mesh quality and accuracy must be maintained while changes in geometry occur [20]. To design the parametric FE model for the tibial insert, the patient-specific TKA was modelled using ABAQUS/CAE 6.13 (Simulia, Providence, RI, USA).

**Figure 2:**
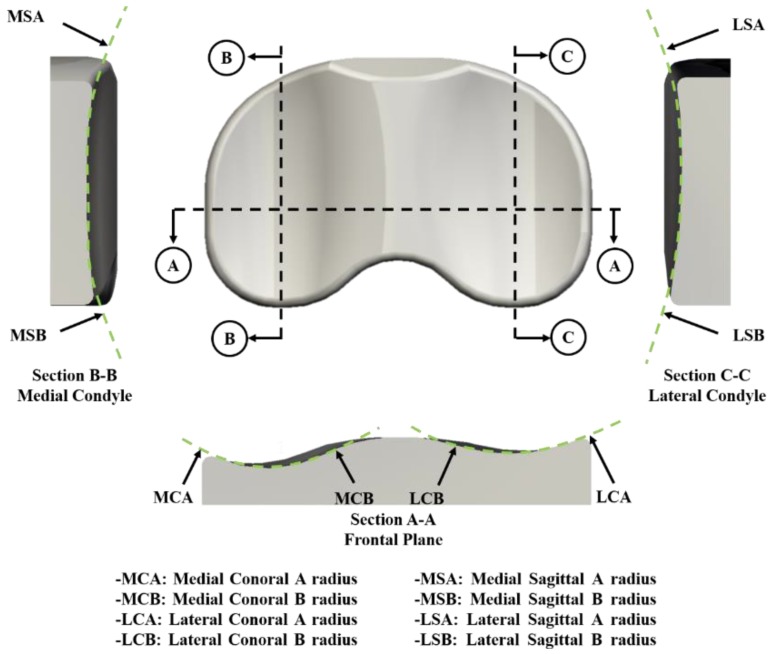
Design variables for the parametric model of tibial insert component.

**Figure 3:**
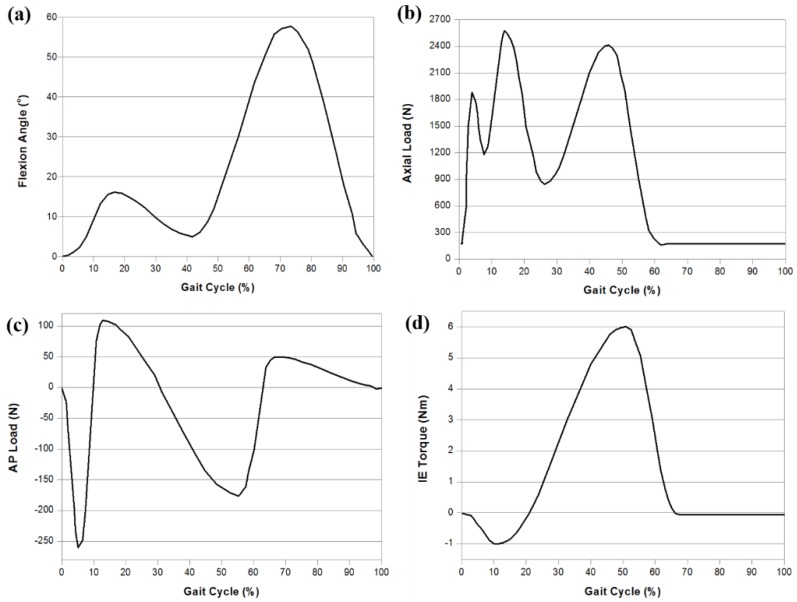
Gait cycle input function for the finite-element (FE) model: (**a**) flexion angle; (**b**) axial load, (**c**) anterior/posterior (AP) load; (**d**) internal/external (IE) torque.

**Figure 4:**
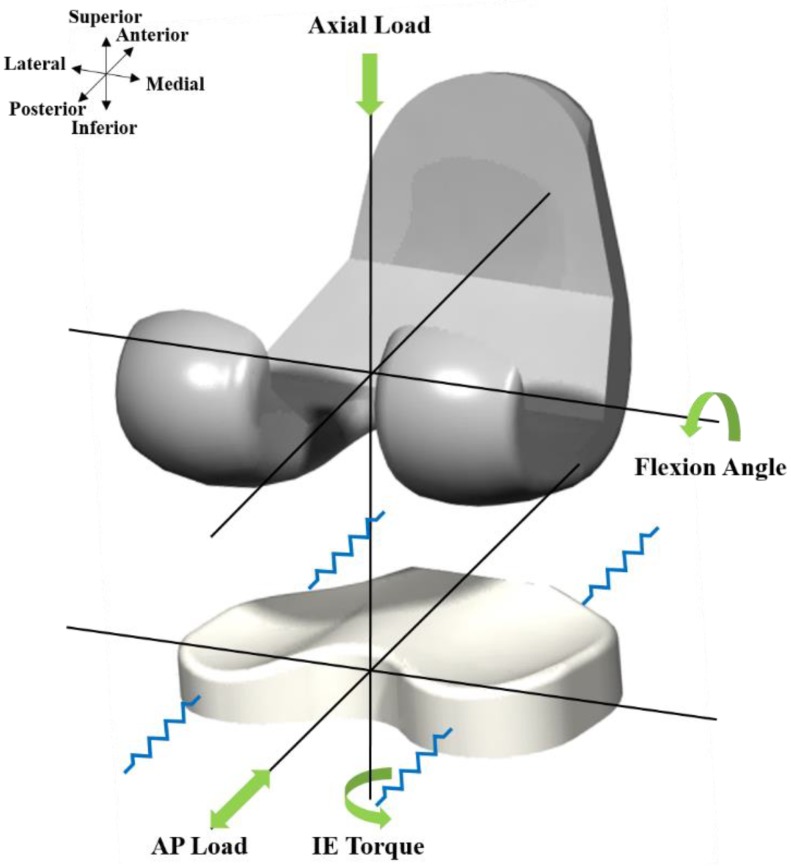
Loading condition of the FE model used in this study.

The tibial insert dimensions for the model were parameterized using eight geometric variables associated with the medial and lateral tibial components (Figure 2). An upper and lower limit for each design variable was determined from the literature [29,30]. The loading and kinematic conditions obtained from experimental studies were used in the FE simulation (Figure 3). The knee simulator model comprises a simulated soft tissue with four springs and a spring gap representing anatomical laxity (Figure 4); the four springs constrain the tibial insert in anterior/posterior (AP) translation and internal/external (IE) rotation. The femoral component and tibial insert are tested with the input profiles of an AP load and IE torque applied to the insert, whereas a flexion-extension angle and an axial force are applied to the femoral component. The axial load, AP translation, and IE rotation are force controlled, whereas the flexion is displacement controlled. The femoral component is constrained in IE, medial-lateral (ML), and AP, and it is free to translate in the inferior-superior (IS) direction to rotate about the frontal and transverse axes to represent varus-valgus (VV) rotation and flexion-extension, respectively. The axial load application is offset towards the medial condyle to reproduce the 60:40 experimental conditions. The distal surface of the tibial insert is supported in the IS direction that is representative of the bonded contact with a rigid tibial tray. The tibial insert is allowed to translate in the AP direction and rotate about a fixed vertical axis located at the center of the tibial condyles to simulate IE rotation. The distal surface of the tibial insert is supported in the IS direction; the insert tilt is constrained; the VV and ML degrees of freedom are unconstrained. The center of rotation for the FE model is directly defined between the medial and lateral condyles. The AP spring translational resistance is 10.4 N/mm, and the IE rotational resistance is 0.30 Nm/deg, which are similar to the experiments.

### 2.3. Wear Prediction of Patient-Specific TKA

To date, no analytical model has been developed that can yield accurate wear predictions. However, a modified version of the Archard’s wear model that evaluates the wear based on a function of contact pressure, contact area, sliding distance, and wear coefficient *k*, is considered a promising model that can deliver a reasonable accuracy if an accurate value of *k* is determined experimentally [31]. The modified Archard’s wear model states that

$$W_{vol}=\iint k\sigma dsdA,$$

where *W_vol_* is the volumetric wear, *k* is the wear coefficient, *σ* is the contact pressure, *s* is the sliding distance, and *A* is the contact area. No wear iteration convergence study was performed. The model is intended for an optimization study, and the evaluation of the single iteration function was a necessary simplification [20]. The model for the wear calculation of the tibial insert was incorporated into the user subroutine VFRICTION that was developed using FORTRAN code. The computed volumetric wear was converted into gravimetric wear using a polyethylene density of 0.93 mm^3^/mg. The wear factor used in this study was estimated considering the average wear factors from TKA and balloon-flat wear tests from a previous study [32].

### 2.4. Design Optimization of Patient Specific TKA

We used the tibial insert wear as the objective function to develop a mathematical problem statement that provides equality and inequality constraints and design variables. Eight design parameters were selected for optimization, and the design optimization was performed using Isight (version 5.9; Dassault Systemes; Vélizy-Villacoublay, France). The design variable limits were selected to enable the maintenance of the model and mesh quality for any particular design for avoiding distorted meshes and possibly inaccurate results. An adaptive remeshing procedure was introduced to simulate the surface optimizations. An adaptive optimization simulation was performed using Python scripts (Stichting Mathematish Centrum, Amsterdam, The Netherlands) to interface with the Abaqus output database. Optimization was conducted using the nondominated sorting genetic algorithm (NSGA-II) that was proposed in the previous study as a suitable method for solving multi-objective problems [33]. The Pareto optimal solutions were evaluated by combining the design factors, and they were labeled as nondominated in the case where no better soluti° to minimize volumetric wear, the multi-objective function was calculated for the optimal design parameters using NSGA-II [34]. The kinematics, wear rate, and volumetric wear of the initial and optimal models were compared. The data in the current study were based on the optimization analysis of 100 trials.

### 2.5. Experimental Wear Simulation

**Figure 5:**
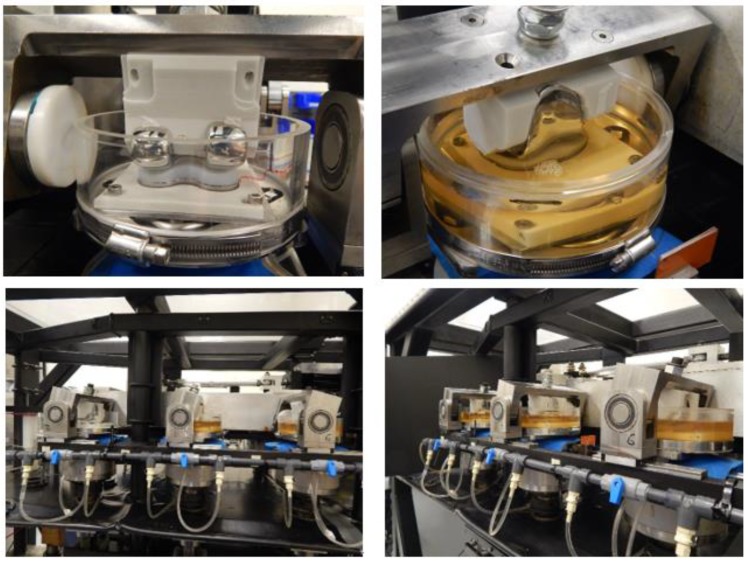
Wear test performed using knee wear simulator with input waveforms; 5 million cycles with a frequency of 1 Hz.

The optimization model was validated experimentally. The femoral component was made of CoCr and manufactured via 3D printing using the selective laser melting process. The tibial insert and components were made of standard PE and Ti6Al4V, respectively, and they were manufactured via machining. Wear testing was conducted using a knee-wear simulator with input waveforms as described above; 5 million cycles were performed with a frequency of 1 Hz (Figure 5). The Hyclone Bovine Calf Serum (63 g/L protein content) at 37 °C was used as a lubricant in all the experiments wherein Bovine serum (25%) was supplemented with EDTA and sodium azide. The pre-soaking period of lubricant was 30 days. The gravimetric measurements were corrected for fluid uptake using a soak control function derived in a separate experimental study. The wear experiments were performed by pausing after each 1 million cycle for the gravimetric measurement of weight loss due to wear, surface-damage measurement using a laser scanner, and bovine serum solution replacement.

## 3. Results

**Figure 6:**
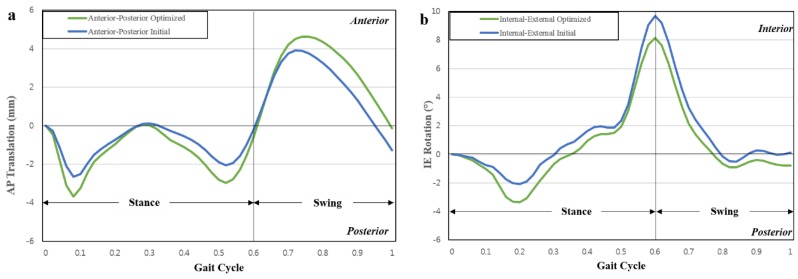
Comparison of kinematics of initial and optimized conformities in customized posterior-stabilized total knee arthroplasty (PS-TKA) under the gait cycle condition: (**a**) AP translation in 0 and 5 million cycles; (**b**) IE rotation in 5 million cycles.

**Figure 7:**
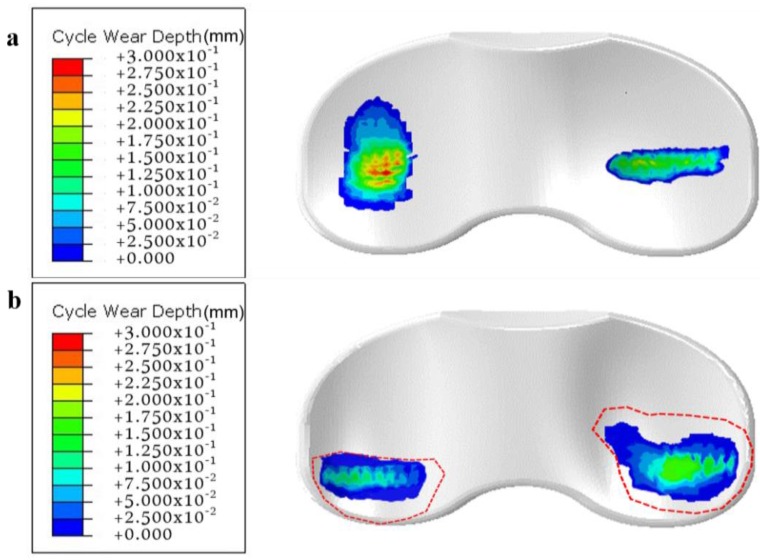
Computational wear contour after 5 million cycles: (**a**) initial model; (**b**) optimal model.

**Figure 8:**
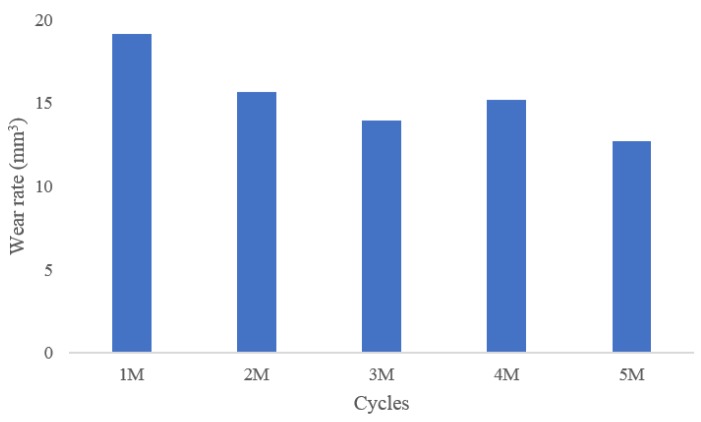
The experimental wear rate in the optimal model per every 1M cycles.

**Figure 9:**
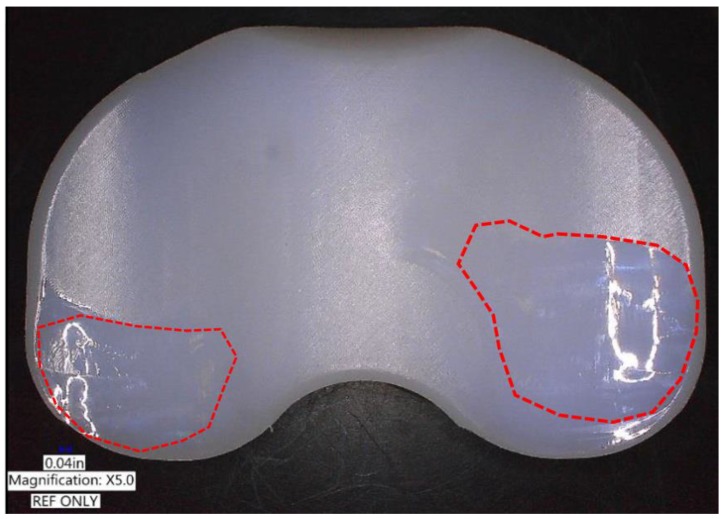
Experimental wear contour of optimal model after 5 million cycles.

Table 1 presents the design variables used in the initial and optimal computational models, and Figure 6 presents the AP and IE kinematic parameters used in the initial and optimal computational models during the gait cycle. In both the models, the internal rotation and anterior translation occurred during the swing phase. The predicted wear rates for the initial and optimal models were 23.2 mm^3^/million cycles and 16.7 mm^3^/million cycles, respectively. The predicted volumetric wear values for the initial and optimal computational models after 5 million cycles were 107.8 mg and 77.6 mg, respectively. The volumetric wear in the optimal model was 28% lower than that in the initial model. Figure 7 illustrates the wear contour of initial and optimal models after 5 million cycles. The wear contour exhibits a deep wear region near the center of the medial side and a shallower wear scar on the lateral side in the initial model. However, the wear was concentrated near the posterior region in both lateral and medial sides of the optimal model. In addition, a deeper wear occurred at the lateral side. The experimental wear rate in the optimal model was 15.3 ± 2.4 mm^3^/million cycles. Figure 8 showed that the experimental wear rate in the optimal model per every 1M cycles. In addition, Figure 9 presents the experimental wear contour in the optimal model for up to 5 million cycles.

Hence, our computational model results were within the standard deviation of the experimental results. In addition, the photographs of the experimental result exhibited a significant similarity with the wear contours of our computed optimal model after 5 million cycles (Figure 9).

## 4. Discussion

The most important result of this study is the wear reduction achieved in the optimal model compared to the initial model. In addition, the developed optimal model was experimentally validated.

Studies based on knee anatomy have reported distinct anatomical differences between the sexes and races. This variations in anatomic sizes can lead to compromise during surgery because it is impossible to maintain implant inventories that precisely match each individual [33]. For each size and design rationale, implant manufacturers have attempted to achieve a better fit at the bone–implant interface. However, potentially adverse events such as femoral component overhang or tibial component under coverage still occur [35]. These issues have led to the development of patient-specific designs with the goals of not only improving the fit of the implant to the knee joint but also restoring normal knee kinematics for each patient. The purpose of patient-specific TKA must be the restoration of the patient’s knee to normal condition as much as possible by correcting any underlying deformity. However, as mentioned previously, the femoral component was designed according to the patient’s anatomy, whereas the tibial insert was constructed using articular geometry derived from the femoral component in current patient-specific TKA technology.

Generally, more conforming designs are favored for decreasing contact stress and structural wear [36], and these designs have proved to improve the stability of the TKA as an additional benefit [37]. Moreover, biomechanical analysis suggested that these highly conforming TKAs may cause over constraint of the knee joint during normal daily activities [38,39]. A retrieval study conforming and less-conforming TKA designs made of modern polyethylene showed that higher conformity tibial inserts caused greater surface damage due to third-body debris trapped within the dished surface, and they were more susceptible to fatigue wear and delamination due to secondary stresses generated from the constraints of the joint [40]. Ardestani et al. quantified the relationships between various geometrical features of a knee implant and their resultant performance metrics [30]. They found that performance metrics are influenced by changes in conformity of the femoral component and tibial insert [30]. Another study demonstrated the trade-off between durability and kinematic performance of TKA [41]. Thus, one of the challenges of TKA design involves determining the optimal conformity conditions considering the above-mentioned potential advantages and disadvantages. Generally, the testing of TKA designs with different conformities has been performed before using knee simulators that include the Stanmore simulator and custom designed simulators [42]. Although in vitro testing is invaluable to evaluate tibial insert materials and geometry, the typical wear tests consume a significant amount of time and incur substantial costs. For these reasons, recent studies have developed computational models of knee simulators to accelerate and improve the implant design process [36,43,44]. Such models generate wear predictions for new TKA designs in a matter of minutes rather than months as in the case of physical wear testing.

Thus, in this study, the tibial insert design was optimized to reduce the wear of patient-specific TKA by applying a parametric computational model and optimization method. Overall, the general trend of the results was comparable to that reported in previous experimental and computational studies based on TKA [17,28,30]. The AP displacement varied in the range of a few millimeters, and IE rotation varied in the range of a few degrees. As mentioned previously, there is a trade-off between the wear performance and kinematics of the tibial insert. Our optimal model showed that kinematics was maintained as wear performance improved compared to the initial model. This is possibly because our study, unlike previous studies, used multi-objective functions for wear and kinematics [20]. There has been considerable discussion on the effect of articular surface conformity on wear. Fregly et al., using a computational model, showed that the increase in conformity offers diminishing returns for reducing TKA wear [44]. In contrast, Abdelgaie et al., also using a computational model, predicted that wear rates for the curved insert were three times higher than those for the flat insert [36]. Although these two studies obtained contrasting results, they both showed that tibiofemoral conformity is an important factor in influencing the wear performance. A previous study reported that the wear-optimized design decreased the radii of curvature of the femoral condyles in the sagittal plane and increased the radii in the frontal plane while reducing the amount of conformity between the femoral condyles and tibial insert [20]. These changes in geometry are expected to alter the articulation leading to the medial-lateral line contact [20]. The smaller product of contact area and sliding distance would offset the effects of the increased contact pressures, thereby reducing the volumetric wear [20]. Our result exhibited complex conformity trends. Our optimal model shows that the medial and lateral sagittal conformities increased and decreased, respectively, compared to the initial model. Our computational results partially contradict the previous computational study that evaluated the effect of sagittal conformity on wear volume [44]. A previous study claimed that to reduce the wear, TKA geometric design efforts aimed at minimizing the wear must focus on sagittal conformity rather than coronal conformity and that at least moderate sagittal conformity is desirable in both compartments [44]. Moreover, in that study, the conformity increased for the coronal plane in the optimal model compared to the initial model. This trend was similar to a previous wear optimization study [20]. This can be because the kinematic increase was considered along with the decrease in wear. This design compares favorably with the one featuring high coronal conformity but less conformity in the sagittal plane [45]. In addition, other studies have shown that increased conformity may have an insignificant effect on tibial insert wear volume because the decreased stress is counteracted by an increased contact area subjected to sliding [46,47].

From the biomechanical perspective, our result showed that kinematics can be maintained as wear decreases through the design optimization method. The femoral component condyles in patient-specific TKA were asymmetric in this study. The characteristics of tibial insert conformity showed that the lateral condyle was less conforming than the highly conforming medial condyle. In the traditional “medial pivot” implants, the medial side is designed to have a “ball-in-socket” articulation [48]. Although the optimal design does not have a perfect “ball-in-socket” articulation conformity, the coronal geometry utilizes a broad radius on both condyles, thereby employing the round-on-round principle that has been shown to reduce contact stress. To maintain the kinematics, the sagittal conformity was controlled. The reduction in the tibial insert wear contributes to longer implant lifetime and a reduced chance of loosening.

There are five limitations in our computational study. First, wear was optimized only for the gait cycle motion. Second, our wear prediction methodology applied a constant wear factor. The future improvements may include different motions such as climbing and squat kinematics, more evaluations of laxity and stability, and the variability present naturally in TKA loading and kinematics due to surgical contributions. Third, we performed wear simulation under idealized conditions. In future study, the malalignment of implants shall be considered. Fourth, patient-specific implants are more expensive than off-the-shelf ones; thus, the cost-effectiveness of their wide use is also debatable. Fifth, viscoelasticity was not considered for the tibial insert. However, previous studies have implemented nonlinear viscoelastic and elastoplastic material models, and they did not detect a significant difference between the wear rates of those models [49]. Finally, the model was developed considering a healthy knee. A perfectly matched implant was designed; however, the biomechanics is not necessarily going to be the same as it was when the patient was healthy because they may lose the reference anatomical dataset.

Despite the limitations, this study has shown that design optimization is an effective approach for TKA design development.

## 5. Conclusions

In conclusion, this study demonstrated that design optimization of patient-specific TKA can improve the wear performance with conserved kinematics. The results obtained in this study provide a potential method of increasing the lifespan of patient-specific TKA. The tibial insert conformity was shown to be a significant factor that affects the wear performance of patient-specific TKA and potentially provides improved clinical outcomes through enhanced design selections. This information can facilitate the future development of patient-specific TKA and be applied to other joint replacement designs. However, future research should be to explore the clinical utility of the findings of this study.

## Figures and Tables

**Table 1:** Design variables: Initial versus optimal design.

	Design Variables (mm)							
	MCA	MCB	MSA	MSB	LCA	LCB	LSA	LSB
Initial	17.00	30.00	38.00	24.00	17.00	32.00	50.00	23.00
Optimal	18.03	33.87	39.15	25.11	18.31	34.32	52.02	24.31

MCA: Medial Conoral A radius; MCB: Medial Conoral B radius; MSA: Medial Sagittal A radius; MSB: Medial Sagittal B radius; LCA: Lateral Conoral A radius; LCB: Lateral Conoral B radius; LSA: Lateral Sagittal A radius; LSB: Lateral Sagittal B radius.

## References

[1] Noble J.W., Moore C.A., Liu N. (2012). The value of patient-matched instrumentation in total knee arthroplasty. J. Arthroplasty.

[2] Brockett C.L., Carbone S., Fisher J., Jennings L.M. (2018). Influence of conformity on the wear of total knee replacement: An experimental study. Proc. Inst. Mech. Eng. H.

[3] Bae D.K., Song S.J., Park M.J., Eoh J.H., Song J.H., Park C.H. (2012). Twenty-year survival analysis in total knee arthroplasty by a single surgeon. J. Arthroplast..

[4] Von Keudell A., Sodha S., Collins J., Minas T., Fitz W., Gomoll A.H. (2014). Patient satisfaction after primary total and unicompartmental knee arthroplasty: An age-dependent analysis. Knee.

[5] Naudie D.D., Ammeen D.J., Engh G.A., Rorabeck C.H. (2007). Wear and osteolysis around total knee arthroplasty. J. Am Acad. Orthop. Surg..

[6] Sharkey P.F., Hozack W.J., Rothman R.H., Shastri S., Jacoby S.M. (2002). Insall Award paper. Why are total knee arthroplasties failing today?. Clin. Orthop. Relat. Res..

[7] White P.B., Ranawat A.S. (2016). Patient-Specific Total Knees Demonstrate a Higher Manipulation Rate Compared to “Off-the-Shelf Implants”. J. Arthroplasty.

[8] Bauwens K., Matthes G., Wich M., Gebhard F., Hanson B., Ekkernkamp A., Stengel D. (2007). Navigated total knee replacement. A meta-analysis. J. Bone Jt. Surg. Am. Vol..

[9] Slover J.D., Tosteson A.N., Bozic K.J., Rubash H.E., Malchau H. (2008). Impact of hospital volume on the economic value of computer navigation for total knee replacement. J. Bone Jt. Surg. Am. Vol..

[10] Kwon O.R., Kang K.T., Son J., Suh D.S., Heo D.B., Koh Y.G. (2017). Patient-specific instrumentation development in TKA: 1st and 2nd generation designs in comparison with conventional instrumentation. Arch. Orthop. Trauma Surg..

[11] Fitz W. (2009). Unicompartmental knee arthroplasty with use of novel patient-specific resurfacing implants and personalized jigs. J. Bone Jt. Surg. Am. Vol..

[12] Steklov N., Slamin J., Srivastav S., D’Lima D. (2010). Unicompartmental knee resurfacing: Enlarged tibio-femoral contact area and reduced contact stress using novel patient-derived geometries. Open Biomed. Eng. J..

[13] Martin G., Swearingen A., Culler S. (2014). Hospital outcomes and cost for patients undergoing a customized individually made TKA vs off-the-shelf TKA. JISRF Reconstr. Rev..

[14] Dalury D.F., Pomeroy D.L., Gorab R.S., Adams M.J. (2013). Why are total knee arthroplasties being revised?. J. Arthroplast..

[15] Kurtz W.B., Slamin J.E., Doody S.W. (2016). Bone Preservation in a Novel Patient Specific Total Knee Replacement. Reconstr. Rev..

[16] Bartel D.L., Bicknell V.L., Wright T.M. (1986). The effect of conformity, thickness, and material on stresses in ultra-high molecular weight components for total joint replacement. J. Bone Jt. Surg. Am. Vol..

[17] Koh Y.G., Son J., Kwon O.R., Kwon S.K., Kang K.T. (2019). Tibiofemoral conformity variation offers changed kinematics and wear performance of customized posterior-stabilized total knee arthroplasty. Knee Surg. Sports Traumatol. Arthrosc..

[18] Koh Y.G., Park K.M., Lee J.A., Nam J.H., Lee H.Y., Kang K.T. (2019). Total knee arthroplasty application of polyetheretherketone and carbon-fiber-reinforced polyetheretherketone: A review. Mater. Sci. Eng. C.

[19] Dargahi J., Najarian S., Amiri S. (2003). Optimization of the geometry of total knee implant in the sagittal plane using FEA. Biomed. Mater. Eng..

[20] Willing R., Kim I.Y. (2009). Three dimensional shape optimization of total knee replacements for reduced wear. Struct. Multidiscip. Optim..

[21] Kang K.T., Koh Y.G., Nam J.H., Jung M., Kim S.J., Kim S.H. (2019). Biomechanical evaluation of the influence of posterolateral corner structures on cruciate ligaments forces during simulated gait and squatting. PLoS ONE.

[22] Koh Y.G., Lee J.A., Lee H.Y., Chun H.J., Kim H.J., Kang K.T. (2019). Anatomy-mimetic design preserves natural kinematics of knee joint in patient-specific mobile-bearing unicompartmental knee arthroplasty. Knee Surg. Sports Traumatol. Arthrosc..

[23] Koh Y.G., Lee J.A., Chung P.K., Kang K.T. (2019). Computational analysis of customized cruciate retaining total knee arthroplasty restoration of native knee joint biomechanics. Artif. Organs.

[24] Kang K.T., Son J., Suh D.S., Kwon S.K., Kwon O.R., Koh Y.G. (2018). Patient-specific medial unicompartmental knee arthroplasty has a greater protective effect on articular cartilage in the lateral compartment: A Finite Element Analysis. Bone Joint Res..

[25] Koh Y.G., Park K.M., Lee H.Y., Kang K.T. (2019). Influence of tibiofemoral congruency design on the wear of patient-specific unicompartmental knee arthroplasty using finite element analysis. Bone Joint Res..

[26] Kim Y.S., Kang K.T., Son J., Kwon O.R., Choi Y.J., Jo S.B., Choi Y.W., Koh Y.G. (2015). Graft Extrusion Related to the Position of Allograft in Lateral Meniscal Allograft Transplantation: Biomechanical Comparison Between Parapatellar and Transpatellar Approaches Using Finite Element Analysis. Arthroscopy.

[27] Kang K.T., Son J., Kim H.J., Baek C., Kwon O.R., Koh Y.G. (2017). Wear predictions for UHMWPE material with various surface properties used on the femoral component in total knee arthroplasty: A computational simulation study. J. Mater. Sci. Mater. Med..

[28] Godest A.C., Beaugonin M., Haug E., Taylor M., Gregson P.J. (2002). Simulation of a knee joint replacement during a gait cycle using explicit finite element analysis. J. Biomech..

[29] Walker P.S., Lowry M.T., Kumar A. (2014). The effect of geometric variations in posterior-stabilized knee designs on motion characteristics measured in a knee loading machine. Clin. Orthop Relat. Res..

[30] Ardestani M.M., Moazen M., Jin Z. (2015). Contribution of geometric design parameters to knee implant performance: Conflicting impact of conformity on kinematics and contact mechanics. Knee.

[31] Archard J., Hirst W. (1956). The wear of metals under unlubricated conditions. Proc. R. Soc. Lond. A.

[32] McGloughlin T.M., Murphy D.M., Kavanagh A.G. (2004). A machine for the preliminary investigation of design features influencing the wear behaviour of knee prostheses. Proc. Inst. Mech. Eng. H.

[33] Deb K., Pratap A., Agarwal S., Meyarivan T. (2002). A fast and elitist multiobjective genetic algorithm: NSGA-II. IEEE Trans. Evol. Comput..

[34] Zhang J., Zhu H., Yang C., Li Y., Wei H. (2011). Multi-objective shape optimization of helico-axial multiphase pump impeller based on NSGA-II and ANN. Energy Convers. Manag..

[35] Patil S., Bunn A., Bugbee W.D., Colwell C.W., D’Lima D.D. (2015). Patient-specific implants with custom cutting blocks better approximate natural knee kinematics than standard TKA without custom cutting blocks. Knee.

[36] Abdelgaied A., Brockett C.L., Liu F., Jennings L.M., Jin Z., Fisher J. (2014). The effect of insert conformity and material on total knee replacement wear. Proc. Inst. Mech. Eng. H.

[37] Luger E., Sathasivam S., Walker P.S. (1997). Inherent differences in the laxity and stability between the intact knee and total knee replacements. Knee.

[38] Sathasivam S., Walker P.S. (1999). The conflicting requirements of laxity and conformity in total knee replacement. J. Biomech..

[39] Fitzpatrick C.K., Clary C.W., Laz P.J., Rullkoetter P.J. (2012). Relative contributions of design, alignment, and loading variability in knee replacement mechanics. J. Orthop. Res..

[40] Wimmer M.A., Laurent M.P., Haman J.D., Jacobs J.J., Galante J.O. (2012). Surface damage versus tibial polyethylene insert conformity: A retrieval study. Clin. Orthop. Relat. Res..

[41] Willing R., Kim I.Y. (2012). Quantifying the competing relationship between durability and kinematics of total knee replacements using multiobjective design optimization and validated computational models. J. Biomech..

[42] DesJardins J.D., Walker P.S., Haider H., Perry J. (2000). The use of a force-controlled dynamic knee simulator to quantify the mechanical performance of total knee replacement designs during functional activity. J. Biomech..

[43] Zhao D., Sakoda H., Sawyer W.G., Banks S.A., Fregly B.J. (2008). Predicting knee replacement damage in a simulator machine using a computational model with a consistent wear factor. J. Biomech. Eng..

[44] Fregly B.J., Marquez-Barrientos C., Banks S.A., DesJardins J.D. (2010). Increased conformity offers diminishing returns for reducing total knee replacement wear. J. Biomech. Eng..

[45] Sathasivam S., Walker P.S. (1994). Optimization of the bearing surface geometry of total knees. J. Biomech..

[46] Bei Y., Fregly B.J., Sawyer W.G., Banks S.A., Kim N.H. (2004). The relationship between contact pressure, insert thickness, and mild wear in total knee replacements. Comput. Model. Eng. Sci..

[47] Essner A., Klein R., Bushelow M., Wang A., Kvitnitsky M., Mahoney O. (2003). The effect of sagittal conformity on knee wear. Wear.

[48] Blaha J.D. (2004). The rationale for a total knee implant that confers anteroposterior stability throughout range of motion. J. Arthroplasty.

[49] Netter J., Hermida J., Flores-Hernandez C., Steklov N., Kester M., D’Lima D.D. (2015). Prediction of wear in crosslinked polyethylene unicompartmental knee arthroplasty. Lubricants.

